# Parametric Optimization of a Truncated Conical Metal Hydride Bed Surrounded by a Ring of PCM for Heat Recovery

**DOI:** 10.3390/ma16083234

**Published:** 2023-04-19

**Authors:** Sofiene Mellouli, Fatma Bouzgarrou, Talal Alqahtani, Salem Algarni, Kaouther Ghachem, Lioua Kolsi

**Affiliations:** 1 Mechanical Engineering Department, College of Engineering, Jazan University, Jazan 45142, Saudi Arabia; 2 Laboratory of Thermal and Energetic Systems Studies (LESTE) at the National School of Engineering of Monastir, University of Monastir, Monastir 5000, Tunisia; 3 Mechanical Engineering Department, College of Engineering, King Khalid University, Abha 62529, Saudi Arabia; 4 Department of Industrial Engineering and Systems, College of Engineering, Princess Nourah bint Abdulrahman University, P.O. Box 84428, Riyadh 11671, Saudi Arabia; 5 Mechanical Engineering Department, College of Engineering, University of Ha’il, Ha’il 81451, Saudi Arabia; 6 Laboratory of Metrology and Energy Systems, National Engineering School of Monastir, University of Monastir, Monastir 5000, Tunisia

**Keywords:** metal hydride, hydrogen, truncated cone, PCM, heat recovery

## Abstract

Metal hydride (MH) hydrogen storage needs an external heat source to release the stored hydrogen. To enhance the thermal performance of MHs, the incorporation of phase change materials (PCM) is a way to preserve reaction heat. This work proposes a new MH-PCM compact disk configuration (i.e., a truncated conical MH bed surrounded by a PCM ring). An optimization method is developed to find the optimal geometrical parameters of the MH truncated cone, which is then compared to a basic configuration (i.e., a cylindrical MH surrounded by a PCM ring). Moreover, a mathematical model is developed and used to optimize the heat transfer in a stack of MH-PCM disks. The optimum geometric parameters found (bottom radius of 0.2, top radius of 0.75 and tilt angle of 58.24) allow the truncated conical MH bed to reach a faster heat transfer rate and a large surface area of higher heat exchange. Compared to a cylindrical configuration, the optimized truncated cone shape enhances the heat transfer rate and the reaction rate in the MH bed by 37.68%.

## 1. Introduction

The conventional hydrogen storage methods include high-pressure gas and low-temperature liquid storage. Among them, solid-state hydrogen storage methods, such as metal hydride (MH) hydrogen storage materials, draw more attention because of their safety, high storage capacity and purification of hydrogen [1]. For many years, hydrogen storage in metal hydrides has been a great environmental and energy success compared to the conventional methods [1,2] with good security features due to the important volumetric energy density and reliability. In the literature, there are some metals that can react with hydrogen and form a metal hydride (MH) [3,4].

Due to the exothermic process that occurs during hydrogen absorption by the metal hydride, heat is emitted and lost to an external fluid. The MH tank then requires an external power source to produce the heat required to desorb the hydrogen from MHs at high temperature and low pressure due to the endothermic nature of the desorption process. The heat is wasted and dispersed during each cycle, which lowers the efficiency of the MH tank. 

In order to understand the complicated coupling of heat and mass transfer in MH tanks, many investigators have conducted experimental and numerical studies [5,6,7,8,9,10,11,12,13]. Researchers have also been interested in enhancing the global hydrogen storage capability by designing new conceptions of HM beds. Several groups of researchers have proposed to integrate fins with different-shaped or heat pipes into the tank to increase the heat transfer area [14,15,16,17,18,19,20,21,22,23,24,25,26,27,28,29]. Others have attempted to maximize the thermal conductivity using metal foams [30,31] and adding expanded graphite [32,33] or a copper matrix [34]. In all of these studies, an external source of heat was needed to power the discharge process. 

In order to prevent this energy loss, researchers developed a useful method that involves reusing the heat reaction by storing it in a phase change material (PCM), which can be used to store and release heat through melting and solidification. This allows storing or releasing the heat necessary for cooling and heating MH beds, respectively. In this context, Garrier et al. [35] created a novel idea for an MH-PCM tank where the exterior tube contains the PCM and the center tube is filled with MgH2 compacted disks. According to the experimental findings, the tank’s storage capacity was significantly increased when compared to a standard tank without PCM. 

Marty et al. [36] suggested an alternative design for an MH tank. It is made up of 71 disks that are compressed magnesium alloy with naturally expanded graphite inside of them. They investigated the dynamic behavior of the reactor under different working conditions and concluded that the PCM surrounding the hydride enhanced the storage capacity. Ben Mâad et al. [37] studied a MH tank integrated with a PCM. Their suggested geometrical arrangement consists of two circular tubes with a center cylinder made of LaNi_5_ and an annular area between the two cylinders filled with PCM. They found that the MH bed was capable of releasing 80% of its hydrogen capacity due to the incorporation of a PCM. After that, they considered the same arrangement and studied the effect of the PCM properties on the MH tank performance [38]. 

The impact of a MH tank design was numerically examined by Mellouli et al. [39]. They studied four MH tank designs: a central tube integrated in the MH tank, hexagonal tubes, spherical shells and cylindrical tubes. The optimal approach, adding cylindrical PCM tubes into the MH bed, improved the charging time by 58.1% when compared to the standard design. Mellouli et al. [40] suggested an additional numerical analysis to assess the integration of a fluid pipe in an MH-PCM tank. It was found that an open heat transfer fluid pipe improved the hydrogen absorption process by 94%.

Indeed, the design of the MH-PCM tank has a substantial effect on its thermal performance to store hydrogen and recover heat. Using a PCM for heat reaction recovery is a promising alternative for improving the MH bed’s performance. Current research focuses on the heat transfer enhancement of reaction beds and high-efficiency heat exchangers for the thermal management of the MH bed. However, the heat transfer within a metal hydride bed is strongly affected by several other internal factors, such as the thermal conductivity of the materials (i.e., the MH bed and PCM), the supplied hydrogen pressure and the mass ratio of PCM to the metal hydride. Few studies were found that dealt with this matter. 

According to the literature review, the location of the PCM in the MH tank is mainly placed around the tank, but this arrangement has a limited heat transfer surface between the PCM and the MH bed. Thus, it is necessary to find a novel configuration that yields the maximum heat transfer surface between the PCM and the MH bed to improve heat transfer for faster hydrogen absorption and desorption rates. More importantly, the constraints on the design of an MH tank are that a larger heat transfer surface is preferred between the MH and the PCM, and on the contrary, the exterior lateral surface of the tank must be minimal on the PCM side to avoid heat losses to the surrounding area, and there is no discussion of this in previous works. 

The truncated conical shell configuration can satisfy the design constraints of the MH-PCM tank. However, a comparison study of various truncated conical shapes is lacking to further reveal the process of better heat transfers and reaction performance.

The objective of this study is to examine the performance of different kinds of MH-PCM disk designs. The base design is a cylindrical MH bed, and the novel design is a MH truncated cone, both of which are surrounded by a PCM ring. Moreover, an optimization approach is developed to find the optimum geometrical parameters of the truncated conical MH bed that satisfy the design constraints of the MH-PCM tank.

## 2. Optimization of the MH Truncated Cone Parameters

**Figure 1:**
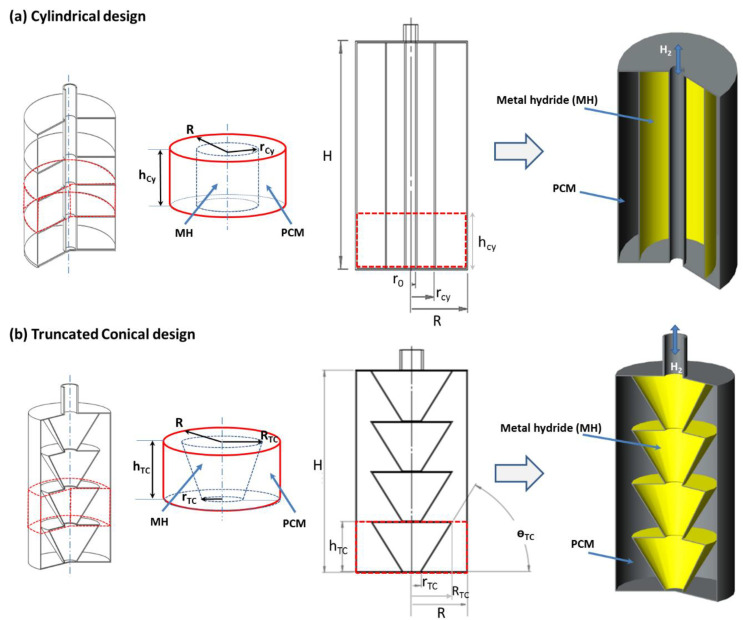
Two kinds of MH-PCM tanks integrated with a stack of compacted disks: (**a**) cylindrical MH bed and (**b**) truncated conical MH bed.

As shown in Figure 1, several MH-compressed disks are stacked in an MH-PCM tank. Two kinds of MH-PCM compact disks are considered. Each MH bed is surrounded by a PCM ring. The base design (Case 1) is a cylindrical MH bed, and the novel design (Case 2) is a truncated conical MH bed. To find the optimum geometrical parameters of the truncated conical MH bed, an optimization approach is developed:

The reaction heat of the hydride is expressed as follows [39]:

(1) $$Q_{MH}=\frac{\omega t(1-\varepsilon )\Delta H_{MH}\rho_{MH}V_{MH}}{M_{H_{2}}}$$

where ωt is the weighted storage capacity of the metal, ε is the porosity, $\Delta H_{MH}$ is the reaction’s enthalpy, $\rho_{MH}$ is the hydride’s density, $V_{MH}$ is the hydride’s volume and $M_{H_{2}}$ is the molar mass of hydrogen.

The heat stored or released by the PCM is expressed as follows [39]:

(2) $$Q_{PCM}=\Delta H_{PCM}\rho_{PCM}V_{PCM}$$

where $\Delta H_{PCM}$ is the latent heat of the PCM, $\rho_{PCM}$ is the PCM density and $V_{PCM}$ is the PCM volume.

In order to store the totality of heat generated by the MH bed, the PCM volume per one kg of MH is stated roughly in a manner that allows the PCM amount to hold all of the heat reaction (i.e., Equation (1) equals Equation (2)); therefore, the volume ratio is expressed as follows:

(3) $$\frac{V_{PCM}}{V_{MH}}=\frac{\omega t(1-\varepsilon )\Delta H_{MH}\rho_{MH}}{\rho_{PCM}M_{H_{2}}\Delta H_{PCM}}=\alpha$$

where α is the volume ratio, which depends on the type of the used materials.

From Equation (3), the PCM volume is expressed as follows:

(4) $$V_{PCM}=\alpha V_{MH}$$

The MH-PCM disk is considered to be a cylindrical configuration for both cases (Case 1 and Case 2). The MH-PCM disk has radius *(R*) and height (*h)*.

The total volume of the MH-PCM disk is expressed as follows:

(5) $$V_{MH-PCM}\;=V_{MH}+V_{PCM}\;\mathrm{or}\;V_{MH-PCM}=\pi R^{2}h$$

For the normalization of the parameters, the radius *(R*) and the height (*h)* of the MH-PCM disk are equal to the unit (i.e., R=h=1). In order to simplify the calculation, the central hydrogen pipe is not considered ($r_{0}=0$).

Combining Equations (4) and (5), the MH-PCM disk volume is expressed as follows:

(6) $$V_{MH-PCM}\;=V_{MH}\left(1+\alpha \right)$$

Combining Equations (5) and (6), the MH bed volume is expressed as follows:

(7) $$V_{MH}\;=\frac{\pi }{\left(1+\alpha \right)}$$

Therefore, from Equations (4) and (7), the PCM volume is expressed as follows:

(8) $$V_{PCM}\;\;=\frac{\alpha \pi }{\left(1+\alpha \right)}$$

Case 1: The base design is a compact MH disk surrounded with PCM, the MH disk has radius ($r_{Cy}$) and height ($h_{Cy}$*),* and the surrounding PCM has radius *(*$R_{Cy}$) as shown in Figure 1a. (The subscript *Cy* refers to the cylindrical configuration).

The MH volume is expressed as follows:

(9) $$V_{MH}{}_{{}_{Cy}}\;=\pi r_{{}_{Cy}}^{2}h_{{}_{Cy}}$$

Combining Equations (7) and (9), the radius of the MH disk is expressed as follows:

(10) $$r_{Cy}\;\;=\sqrt{\frac{1}{\left(1+\alpha \right)}}$$

The maximum lateral heat exchange surface area with the PCM jacket is:

(11) $$A_{Cy}\;\;=2\pi h_{Cy}\sqrt{\frac{1}{\left(1+\alpha \right)}}$$

Case 2: The novel MH disk is a truncated cone that is surrounded by a cylindrical PCM shell as shown in Figure 1b. The PCM cylinder inscribes the MH truncated cone such that the heights of the MH truncated cone and the PCM cylinder are the same, and then the volume of the PCM cylinder and the volume of the MH truncated cone are expressed as given below: (the subscripts TC refer to the truncated cone).

From Equation (4), the PCM volume is expressed as follows:

(12) $$V_{PCM_{TC}}\;=\alpha V_{MH,}{}_{TC}$$

The MH volume is expressed as follows:

(13) $$V_{MH,}{}_{TC}\;=\frac{\pi h_{TC}}{3}\left(R_{TC}^{2}+R_{TC}r_{TC}+r_{TC}^{2}\right)$$

where $h_{TC}$ is the height of the MH disk, $r_{TC}$ is the radius of the bottom and $R_{TC}$ is the radius of the top.

For a cylindrical configuration of the MH bed disk, it can hold a certain volume of MH bed Equation (7); however, if the sides are slanted, it will hold more MH bed. If the sides are sloped out, then the top of the disk has a larger radius, which yields more heat exchange surface area ($A_{TC}\geq A_{Cy}$) with the PCM domain, and so, if the same amount of MH bed is used, less height in the truncated cone disk results (i.e., $h_{TC}\leq h_{Cy}\;$). In order to compare the performance of the two cases, the same materials are taken (i.e., $V_{MH,}{}_{TC}=V_{MH,}{}_{Cy}\;$).

The base surface area of the MH-PCM cylinder (Case 1) equals the minimal surface area of the truncated cone (Case 2). The methodology entails considering a planar circle with a radius R=1. The surface area of the disk is $A=\pi R^{2}\;$. This flat disk slug can be bent into a frustum shape, which has sides with various angles C and a cylindrical base at the bottom. The height (h) is varied depending on the frustum’s top radius (r). Each configuration has the same minimum surface area (bottom plus sides), but the height will differ greatly based on the radius ratio.

(14) $$A_{TC,\min}\;=\pi r_{TC}^{2}+\pi \left(R_{TC}+r_{TC}\right)\sqrt{{\left(R_{TC}-r_{TC}\right)}^{2}+h_{TC}^{2}}$$

However, our objective is to reach the maximum heat exchange surface area with the PCM domain. When the truncated cone MH disks are stacked, the heat exchange surface area with PCM is increased (sides + top-bottom) and reaches a maximum ($A_{TC,\max}$). The maximum surface area is reached when the bottom radius $r_{TC}=o$ (i.e., when the shape is a cone). Hence, the heat exchange surface area has limits where it is greater than that of Case 1 ($A_{Cy}$) and lower than the maximum surface area ($A_{TC,\max}$).

(15) $$A_{Cy}\;<A_{TC}<A_{TC,\max}$$

(16) $$A_{TC,\max}\;=2\pi R^{2}$$

Combining Equations (11) and (16) and considering that the size of the MH-PCM compacted disk (*R* = *h* = 1) is normalized, the normalized surface area is limited as follows:

(17) $$\frac{1}{\sqrt{1+\alpha }}\;<{\bar{A}}_{TC}<1$$

The maximal MH bed volume can be calculated for all feasible top and bottom radius ($R_{TC}$,$r_{TC}$) values if the minimal surface area of the frustum is maintained at that of the starting disk.

Combining Equations (13) and (14), the height of the MH disk is expressed as follows:

(18) $$h_{TC}\;=\sqrt{\frac{A_{TC}-\pi r_{TC}^{2}}{\pi (R_{TC}+r_{TC})}-{\left(R_{TC}-r_{TC}\right)}^{2}}$$

Combining Equations (13) and (18) yields the following expression of the MH bed volume:

(19) $$V_{MH,}{}_{TC}\;=\frac{\pi }{3}\left(R_{TC}^{2}+R_{TC}r_{TC}+r_{TC}^{2}\right)\sqrt{\frac{1-r_{TC}^{2}}{\left(R_{TC}+r_{TC}\right)}-{\left(R_{TC}-r_{TC}\right)}^{2}}$$

The MH bed’s normalized volume (Case 2) is limited to that of Case 1, which corresponds to Equation (14). Hence, the normalized volume is expressed as follows:

(20) $${\bar{V}}_{MH,}{}_{TC}=\frac{V_{MH,}{}_{TC}}{V_{MH,}{}_{Cy}}$$

In Equation (19), each radius ($R_{TC}$ and $r_{TC}$) has a possible range of 0 to 1. The MH bed is a flat disk with no volume if either the top or bottom radius is set to 1. The MH bed is a cone when the bottom radius $r_{TC}=0$.

The radii ($R_{TC}$ and $r_{TC}$) are varied from 0 to 1, and then, for each maximum MH bed volume (${\bar{V}}_{MH,}{}_{TC}=1$), the surface area is calculated (${\bar{A}}_{TC}$). The optimal parameters ($r_{TC}$, $R_{TC}$, $h_{TC}$ and $\theta_{TC}$) of the truncated cone MH bed are found in the results section. After that, the novel configuration with the found optimum parameters (Case 2) is compared to the base configuration (Case 1) via the numerical simulations.

## 3. Mathematical Model

### 3.1. Governing Equations

**Figure 2:**
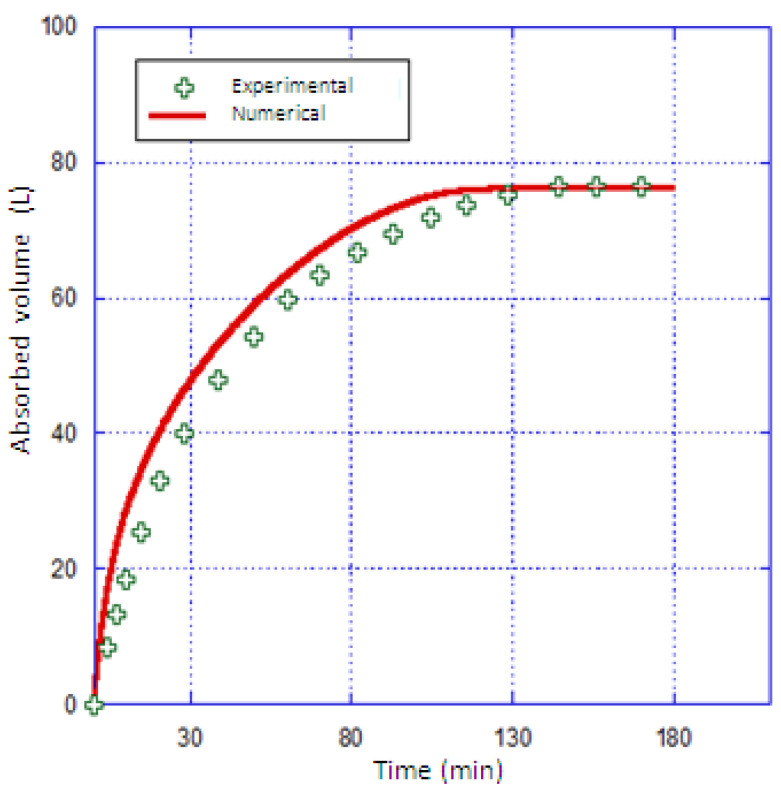
Validation of the model.

The considered MH-PCM tank contains a stack of four compacted disks (Figure 2). The macroscopic differential equations for the MH-PCM tank are developed with consideration of the following assumptions [38,39,40]:

− Regarding the axis symmetry of the MH-PCM stacked disks, only half of the physical domain is simulated.
− The hydrogen pressure is uniform.
− The local thermal equilibrium is considered.
− The properties of the materials are constant.
− The natural convection effect in the PCM is neglected [38,39,40,41].

Considering these assumptions, the following equations are provided to describe heat and mass transfer within the MHT-PCM tank:

(21) $$\frac{\partial }{\partial t}\left(f_{\varphi }\varphi \right)=\xi_{\varphi }\left[\frac{\partial^{2}\varphi }{\partial^{2}z}+\frac{1}{r}\frac{\partial }{\partial r}\left(r\frac{\partial \varphi }{\partial r}\right)\right]+S_{\varphi }$$

The expressions of the different elements used in this equation are given for the MH bed and the PCM ring in Table 1.

The rate of hydrogen can be expressed as follows:

(22) $$m=\rho_{Mg}\omega t(1-\varepsilon )\frac{dX}{dt}$$

The kinetic reaction for hydrogen absorption is as follows [39,40]:

(23) $$\frac{dX}{dt}=\left\{\begin{array}{c} k_{0}\frac{X-1}{2\ln(1-X)}\left(\frac{P}{P_{eq}}-1\right)e^{-Ea/RT}\;P_{eq}\langle P\langle 2P_{eq}\\ k_{0}(1-X)\left(\frac{P}{P_{eq}}-1\right)e^{-Ea/RT}\;P\rangle 2P_{eq} \end{array}\;\right.$$

The equilibrium pressure expression is as follows:

(24) $$Ln\frac{P_{eq}}{P_{ref}}=\frac{\Delta H}{RT}-\frac{\Delta S}{R}$$

The PCM melted fraction is expressed as follows [39]:

(25) $$F=\left\{\begin{array}{l} 0\;\;\;\;if\;\;\;\;T\leq T_{sol}\\ 1\;\;\;\;if\;\;\;\;T\geq T_{liq}\\ \frac{\left(T-T_{sol}\right)}{\left(T_{liq}-T_{sol}\right)}\;if\;\;T_{sol}<T<T_{liq} \end{array}\;\right.$$

### 3.2. Initial and Boundary Conditions

The temperature and the pressure are both uniform.

(26) $$P=\;P_{0},\;T=\;T_{0}=T_{m}$$

For the symmetry boundary and insulated boundary:

(27) $$\nabla T.\vec{n}=0$$

where $\vec{n}$ is the normal vector to the relative wall.

For the inter-domains MH/PCM:

(28) $$\lambda_{PCM}\nabla T_{PCM}.\vec{n}=\lambda_{MH}\nabla T_{MH}.\vec{n}$$

### 3.3. Validation Model

**Figure 3:**
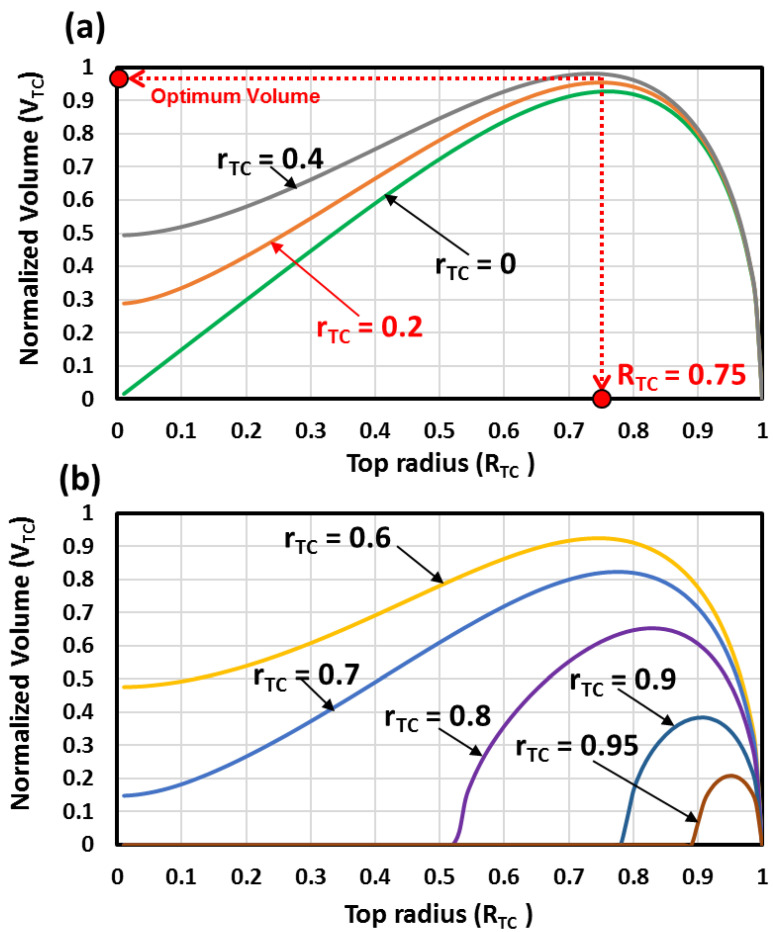
The MH bed volume variations against the top radius and the bottom radius (**a**) for $0\leq r_{TC}\leq 0.4$ and (**b**) for $0.6\leq r_{TC}\leq 0.95$.

Using Fluent 6.3.26, the mathematical model was numerically solved. The numerical results were compared to the experimental results reported by Chaise et al. [35]. Figure 3 depicts the expected and calculated hydrogen storage capacities. The simulation findings and experimental data line up well [35].

## 4. Results and Discussion

The parametric optimization approach consists of determining the optimum geometrical parameters of the truncated conical MH bed that yield faster heat transfer compared to the basic cylindrical case. The design constraints of the truncated conical MH bed are listed as follows:

(i) The reference MH-PCM disk corresponds to the case, where R=h=1.
(ii) The MH bed volume must be the same for all the cases (i.e., $V_{MH}=\pi r_{cy}^{2}h≅\pi$)
(iii) The heat exchange surface area must be greater than that of the cylindrical case ($A_{\min}=2\pi r_{cy}h\approx \pi$).
(iv) The maximum top radius $R_{TC}=1$.
(v) The volume of the surrounded PCM ring must be the same for all the cases (i.e., corresponds to Equation (12)).

Considering the constraints of the design, several geometrical parameters of the truncated conical MH bed were tested. The amounts of the metal hydride bed and the PCM were kept the same for all the cases. For the normalization of the parameters, the radius *(R*) and the height (*h)* of the MH-PCM disk were kept equal to the unit (i.e.,R=h=1). In order to simplify the calculation, the central hydrogen pipe was not considered ($r_{0}=0$).

Using Equation (19), the radii ($R_{TC}$ and $r_{TC}$) were varied to achieve the desired MH bed volume (${\bar{V}}_{MH,}{}_{TC}=1$) and the maximum surface area (${\bar{A}}_{TC}$). The optimal parameters ($r_{TC}$, $R_{TC}$, $h_{TC}$ and $\theta_{TC}$) of the truncated cone MH bed were deduced afterwards.

The bottom radius ($r_{TC}$) was kept to constant values (0, 0.2, 0.4, 0.6, 0.8, 0.9 and 0.95), and the top radius ($R_{TC}$) varied from 0 to 1. The obtained curves of the MH bed volume are depicted in Figure 3. When the bottom radius was kept at zero ($r_{TC}=0$), and the top radius ($R_{TC}$) varied from 0 to 1, the shape of the MH bed was a cone. The obtained curve of the MH bed volume is depicted in Figure 3.

**Figure 4:**
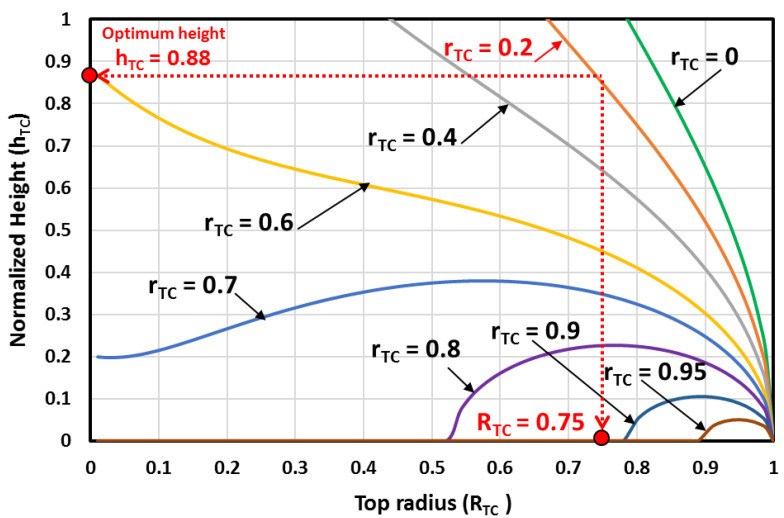
The MH bed height variations against the top radius and the bottom radius.

**Figure 5:**
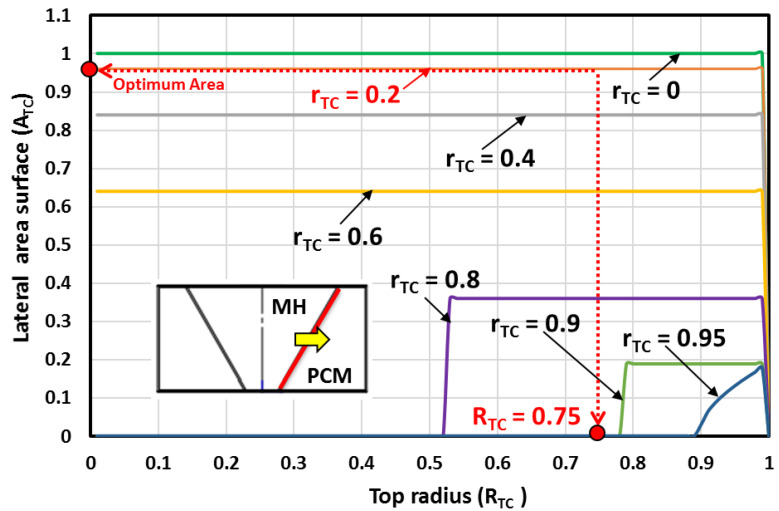
The lateral surface area for different top and bottom radii.

**Figure 6:**
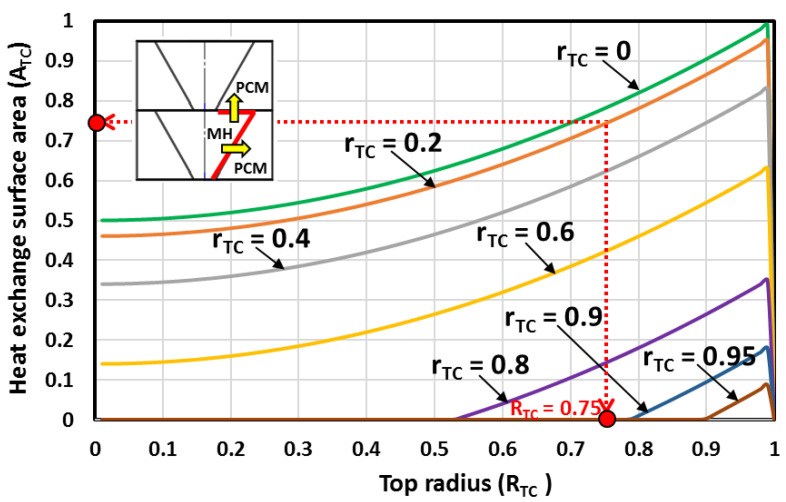
The heat exchange surface area between two MH-PCM stacked disks for different top and bottom radii.

The MH beds are all truncated cones shape when the bottom radius is maintained at $r_{TC}\neq 0$. The MH bed has no volume when $R_{TC}=1$ (this is simply a planar disk with no MH bed volume), and it is undefinable when $R_{TC}=0$. From Figure 3, the optimal MH bed volume is determined, and the corresponding height is deduced from Figure 4. Consequently, the heat exchange surface area is determined for each optimal volume from Figure 5 and Figure 6.

The MH bed volume variations against the top and bottom radii are depicted in Figure 3. From this figure, it is observed that there is a maximum volume for the MH bed. Under the defined design constraints and for the desired MH bed volume, the higher MH bed volumes are achieved when the top radius is near ($R_{TC}\approx 0.75$). It is observed that the higher MH bed volumes are reached when the bottom radius $0\leq r_{TC}\leq 0.4$ (see Figure 3a). However, if the bottom radius $0.6\leq r_{TC}\leq 95$, lower volumes are observed (see Figure 3b).

Under the defined design constraints, the desired optimum MH bed volume is reached when the top radius is near $R_{TC}=0.75$. Clearly, the desired MH bed volume is near the optimum value when the top radius is $r_{TC}=0.4$, $r_{TC}=0.2$ or $r_{TC}=0$. However, in real situations, a hydrogen filter pipe is inserted centrally in the MH bed. Therefore, the MH bed cone shape ($r_{TC}=0$) is dropped, and only the truncated conical shapes are retained. From Figure 5, it is observed that the larger lateral surface area is reached when the top radius is $R_{TC}=0.75$ and when the bottom radius is $r_{TC}=0.2$. Then, the case with $r_{TC}=0.4$ is dropped, and only the case with $r_{TC}=0.2$ is retained.

Figure 4 shows the MH bed height variations against the top radius and the bottom radius. The figure is used to determine the height of the MH-PCM disk corresponding to the selected parameters. For values of the bottom radius near 0, as the top radius gets larger, the truncated conical MH bed gets longer and has a higher height. For values of the bottom radius near 1, as the top radius gets larger, the truncated conical MH bed becomes flatter and has a lower height.

The lateral surface for different top and bottom radii is depicted in Figure 5. For values of the bottom radius in the range of 0 and 0.4, as the bottom radius gets smaller, the lateral surface area gets larger. Similarly, as the bottom radius gets larger, the MH bed becomes flatter and has less lateral surface area than the fixed minimum surface area value.

The heat exchange surface area between two MH-PCM stacked disks is depicted in Figure 6 for different top and bottom radii. Under the defined design constraints, the higher surface area is reached when the MH bed shape is a cone ($r_{TC}=0$). However, in real situations, a hydrogen filter tube (with a radius $r_{o}<<r_{TC}$) is inserted in the center of the MH bed. Therefore, the MH bed cone shape is dropped, and only the truncated conical shapes are retained. It is observed that the larger heat exchange surface area between two MH-PCM stacked disks is reached when the bottom radius is $r_{TC}=0.2$ and when the top radius is $R_{TC}=0.75$.

**Figure 7:**
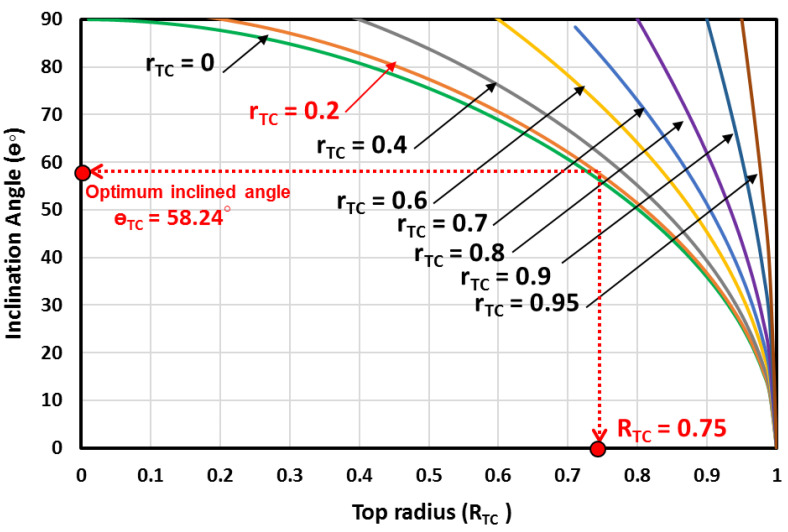
The inclination angle against the top radius and the bottom radius.

The inclination angle of the truncated cone shapes is calculated for different top and bottom radii. Figure 7 depicts the evolution of the inclination angle (θ) against the top radius ($R_{TC}$) and the bottom radius ($r_{TC}$). It is seen that, for all the cases where the top radius is equal to the bottom radius, the shape of the MH bed is a cylinder, and the inclined angle is $\theta =90^{ο}$ as shown in Figure 7. The figure is used to determine the optimum inclination angle, which is $\theta =58.24^{ο}$, which corresponds to a bottom radius of $r_{TC}=0.2$ and a top radius of $R_{TC}=0.75$.

In order to confirm the proposed optimization approach and to prove that the developed equations are able to adequately optimize the truncated conical MH bed, a mathematical model was created. Two types of tanks were simulated with four MH-PCM stacked disks integrated in each tank. The geometrical parameters of the MH-PCM stacked disks are listed in Table 2.

**Figure 8:**
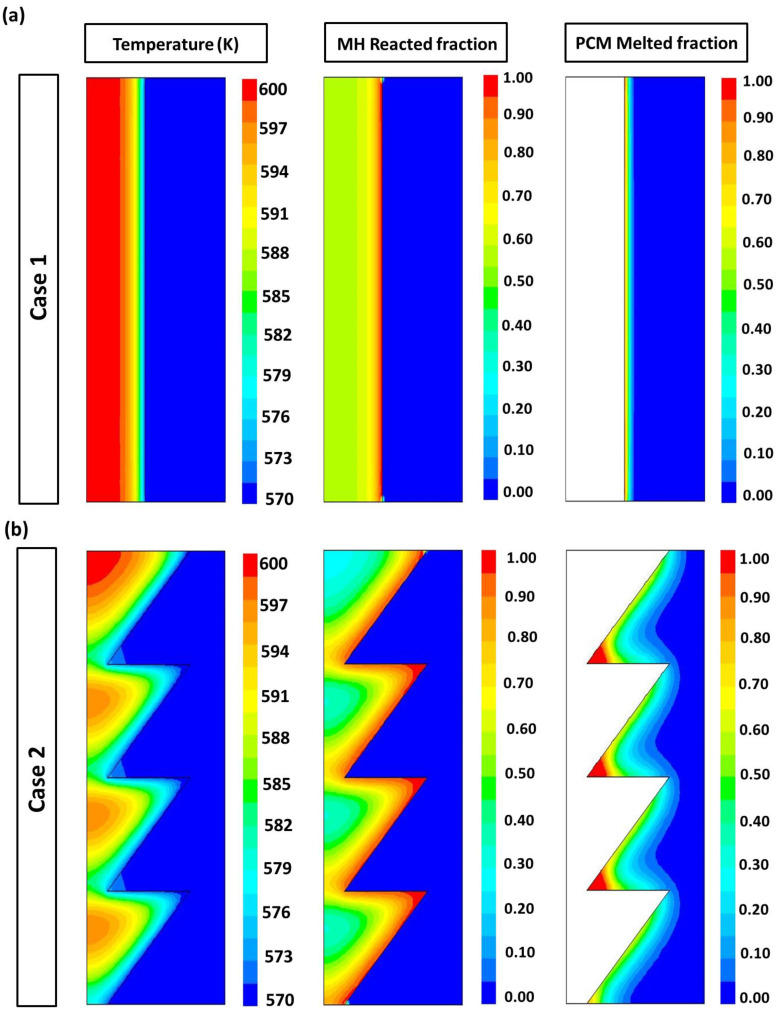
Contours of the temperature, the reacted fraction and the PCM melted fraction within two kinds of MH-PCM stacked disk configurations: (**a**) cylindrical MH bed and (**b**) truncated conical MH bed.

The contours of the temperature, the reacted fraction and the PCM melted fraction within the two kinds of MH-PCM tanks are shown in Figure 8. The colors are the temperature, the reacted fraction and the melted PCM fraction, with the colors coded as follows: Red = high-value parameters. Green = mid-value parameters. Blue = low-value parameters. The temperature of the MH bed increases near the center and decreases near the PCM ring wall due to the heat transferred and stored in the PCM. From this figure, it is also seen that the temperature decreases rapidly for the case with a truncated conical MH bed. 

The PCM temperature rises to the melting temperature, and the melting process starts. With the heat transfer from the frustum MH bed to the PCM ring, the reacted fraction increases from the inter-boundary wall to the center of the MH bed, while the front of the melting fraction increases from the inter-wall to the external wall of the PCM-surrounded ring. According to Figure 8, the heat transfer rate, the hydrogen reaction rate and the PCM melting rate are faster in the MH bed with a truncated conical shape compared to the MH bed with a cylindrical case. The hydrogen reaction rate of the truncated configuration is about two times that of the cylindrical structure, owing to the larger heat transfer area. Figure 8 shows that the PCM melting process is more uniform in the cylindrical configuration (Case 1). 

This is because the thermal resistance of MH is smaller than that of PCM, and there is no obvious temperature gradient in the MH bed; thus, the melted fraction and the reacted fraction are more uniform in Case 1. However, in the truncated configuration (Case 2), PCM begins to melt faster from the inter-wall to the external wall, storing heat in the MH bed.

The geometrical parameters for the calculated truncated conical MH-PCM disks, which were found in the aforementioned section, are listed in Table 3.

**Figure 9:**
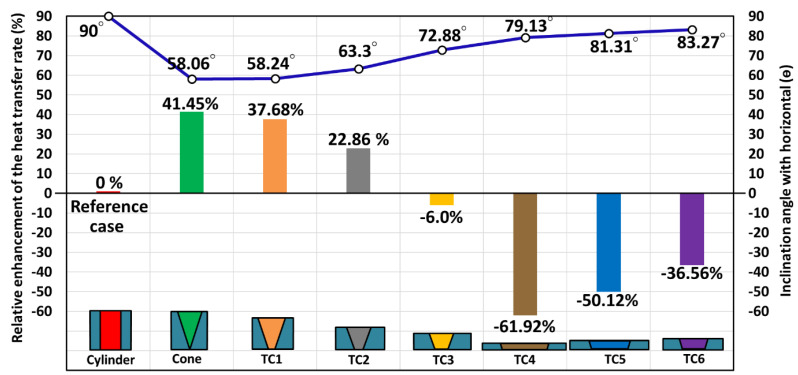
Relative enhancement of the heat transfer rate in the truncated conical MH bed (Case 2) compared to the basic cylindrical Case 1 (i.e., different geometric parameters are tested according to Table 3).

The relative enhancement of the heat transfer rate in the few truncated conical MH disks (Case 2) is compared to the basic cylindrical Case 1 (i.e., different geometric parameters are tested according to Table 3) and is depicted in Figure 9.

The heat transfer rate of the MH-PCM tank is improved from 22.86% to 41.45% by integrating the optimized truncated conical MH bed. According to Figure 9, for the flatter and thinner MH-PCM disks, the heat transfer rate is lower due to the lower surface area and deficient heat transfer between the MH bed and PCM, where the deficiency ranges from −36.56% to −61.92.

Finally, it was concluded that the truncated conical MH bed geometrical parameters must be optimized to avoid the delay of the heat transfer rate and the hydrogen absorption/desorption process in the metal hydrides.

## 5. Conclusions

We proposed a novel MH-PCM compacted disk design (i.e., a truncated conical MH bed surrounded by a PCM ring). An optimization method was developed and used to find the optimum geometrical parameters of the truncated conical MH bed. The proposed MH-PCM disk was compared to a basic disk configuration (i.e., a cylindrical MH bed surrounded by a PCM ring). Moreover, a mathematical model was developed and used to optimize the heat transfer in a MH-PCM stacked disk. The following are our major conclusions:

(i) The results confirm that the proposed approach and the developed equations are able to adequately estimate the optimum geometrical parameters of the truncated conical MH bed.
(ii) The novelty of the study is to optimize the design of the truncated conical MH bed surrounded with a PCM ring for heat reaction recovery. This idea was proposed as a solution to enhance the heat transfer rate and reduce the duration of the hydrogen absorption and desorption in the MH tank.
(iii) The optimum geometrical parameters of the truncated conical MH bed are the bottom radius of $r_{TC}=0.2$, the top radius of $R_{TC}=0.75$ and the optimum inclination angle of $\theta =58.24^{ο}$. These parameters allow the yield of a larger heat exchange surface area between two MH-PCM stacked disks and, consequently, a faster heat transfer rate.
(iv) The heat transfer rate of the MH-PCM tank was improved 37.68% by integrating the truncated conical shape MH bed.
(v) A similar approach was proposed by Yang et al. [42], where the PCM was sandwiched between two layers of MH beds, and they found that the PCM sandwiched structure had a larger heat transfer area compared with the surrounding cylindrical structure. However, in this paper, the obtained results found that the surrounding truncated structure had a larger heat transfer area compared with the sandwiched structure.
(vi) The new MH-PCM truncated conical disk is an effective tank configuration. Nevertheless, selecting the optimal geometrical parameters, low-cost materials and suitable operating conditions is necessary for effective operation in next-generation, large-scale metal hydride tanks.

## Figures and Tables

**Table 1:** The expression of the different terms of Equation (21) [39,40].

Equations	Domain	φ	$f_{\varphi }$	$\xi_{\varphi }$	$S_{\varphi }$
Energy	MH bed	T	$\varepsilon \rho_{H_{2}}C_{pH_{2}}+\left(1-\varepsilon \right)\rho_{Mg}C_{pMg}$	$\varepsilon \lambda_{H_{2}}+\left(1-\varepsilon \right)\lambda_{Mg}$	$m\left(\frac{\Delta H_{MgH_{2}}}{M}-Cp_{H_{2}}(T-T^{0})\right)$
	PCM	H	$\rho_{PCM}\;$	$\lambda_{PCM}$	$\rho_{PCM}C_{P_{PCM}}\frac{dF}{dt}$
Continuity	MH bed	1	$\left(1-\varepsilon \right)\rho_{Mg}$	0	−m

**Table 2:** The different parameters of the MH tanks.

Geometrical Parameters					
Configuration	*r*	*R*	*R_PCM_*	*H*	*ɵ*
Cylindrical	0.476	-	1	4	90
Truncated shell	0.2	0.75	1.17	3.55	58.24

**Table 3:** The geometric parameters of the MH-PCM disk for different cases.

Parameters	Cylindrical	Cone	Truncated Cone MH Bed					
			TC1	TC2	TC3	TC4	TC5	TC6
r	0.472	0	0.2	0.41	0.6	0.9	0.8	0.7
R	0.472	0.747	0.75	0.74	0.75	0.95	0.85	0.75
h	1	1.198	0.888	0.656	0.487	0.260	0.327	0.423
$R_{PCM}\;$	1	1.011	1.174	1.366	1.586	2.169	1.935	1.700
θ	90	58.066	58.249	63.304	72.889	79.134	81.317	83.273
$A_{Sides}\;$	2.965	3.312	3.117	2.652	2.161	1.540	1.716	1.943
$A_{Ex}\;$	2.965	5.064	4.758	3.844	2.797	1.831	1.975	2.171

## Data Availability

Not applicable.
